# Demographic and mortality analysis of hospitalized children at a referral hospital in Addis Ababa, Ethiopia

**DOI:** 10.1186/s12887-016-0709-4

**Published:** 2016-10-21

**Authors:** J. A. Bohn, B. M. Kassaye, D. Record, B. C. Chou, I. L. Kraft, J. C. Purdy, K. A. Hilton, D. A. Miller, S. Getachew, A. Addissie, J. A. Robison

**Affiliations:** 1 Department of Pediatrics, Division of Pediatric Emergency Medicine, University of Utah School of Medicine, 30 N. 1900 E., Salt Lake City, UT USA; 2 Addis Ababa University School of Medicine, Addis Ababa, Ethiopia; 3 Pacific Northwest University of Health Sciences, 111 University Parkway, Yakima, WA USA; 4 Department of Preventive Medicine, Addis Ababa University School of Public Health, Addis Ababa, Ethiopia

**Keywords:** Hospital mortality, Child health, Global health, Pediatric emergency

## Abstract

**Background:**

Global childhood mortality rates remain high. Millennium Development Goal 4 focused efforts on reducing rates by two-thirds between 1990 and 2015. In Ethiopia, child mortality rates dropped 71 % from 1990 to 2015, however it is estimated that 184,000 Ethiopian children die each year. There is limited information about pediatric hospital admissions in Ethiopia. Our aims were to examine the temporal relationship of mortality to admission, describe the demographics, and identify cause mortality of children admitted to the Zewditu Memorial Hospital (ZMH).

**Methods:**

A four-year retrospective review of pediatric admissions was conducted at the pediatric emergency room and pediatric hospital ward at ZMH in Addis Ababa, Ethiopia. Admission entries from 2011–2014 of children age 29 days-14 years were reviewed. Age, gender, admission date, disease classification, discharge status and date were obtained. Patient gender was compared using Chi-square analysis. A descriptive analysis was used for age and cause mortality.

**Results:**

A total of 6866 patient entries were reviewed. The proportion of admissions younger than age 5 was 0.747 (95 % CI 0.736-0.757). Overall mortality was 0.042 (95 % CI, 0.037-0.047). The proportion of recorded deaths occurring within 2 days of admission was 0.437 (95 % CI 0.380-0.494). The proportion of male admissions was significantly higher than female admissions in all age groups (male 0.575, *p* < 0.0001, 95 % CI 0.562-0.586). The main causes of mortality were pneumonia (0.253, 95 % CI, 0.203-0.303), severe acute malnutrition (0.222, 95 % CI 0.174-0.27), HIV/AIDS-related complications (0.056, 95 % CI 0.029-0.083), spina bifida (0.049, 95 % CI 0.024-0.074), and hydrocephalus (0.045, 95 % CI 0.021-0.069).

**Conclusions:**

Our study revealed a lower mortality rate than previously reported in Ethiopia. Despite this, 44 % of pediatric hospital mortality occurred early during hospitalization, higher than reported at other Ethiopian hospitals. This adds further evidence that systematic efforts should be dedicated to improve pediatric emergency care. Admissions included 58 % male patients, similar to other reports in Ethiopia implying that this may be a nation-wide phenomenon. The observed disparity may be due to societal factors regarding care-seeking behaviors or male predilection for respiratory illness warranting further investigation. Cause mortality patterns were similar to reports in analogous settings.

## Background

Despite substantial progress reducing child deaths, global childhood mortality rates remain high [1]. Millennium Development Goal (MDG) 4 focused efforts on reducing these rates by two-thirds between 1990 and 2015. During this time, mortality rates of children under age five have dropped 53%, from 91 deaths per 1,000 live births in 1990 to 43 deaths per 1,000 in 2015. Despite this decrease, about 16,000 children under five years of age still die every day [1, 2], and the WHO African region still has a child mortality rate of 92 deaths per 1,000 live births which is more than 15 times the average mortality of developed regions, and the highest of all WHO regions [2].

Out of 60 countries characterized as having a high childhood mortality (at least 40 deaths per 1,000 live births in 2013), Ethiopia is among eight that have actually achieved MDG 4 [2]. With a childhood mortality rate decrease of 71%, from 205 deaths per 1000 live births in 1990 to 59 deaths per 1000 live births in 2015, Ethiopia is demonstrating that lowering under-five mortality is possible in low-income counties [1, 2]. This goal was achieved through a combination of factors including: policy changes, technological advancements, and the delivery of improved health services to the rural population, largely through the Health Extension Program (HEP) [3].

The HEP was launched by the Ethiopian Federal Ministry of Health in 2003 and is comprised of Health Extension Workers (HEW) trained to promote health knowledge and skill in rural areas. The program has improved the education of households around disease prevention, family health and hygiene. By 2010, approximately 30,000 HEWs were serving in a majority of villages in rural areas and HEP had become the country’s major health program [4]. Yet despite these significant achievements, it is estimated that about 184,000 Ethiopian children die each year before reaching the age of five [1, 2, 5].

While great strides have been made in the public health sector in Ethiopia, less is known about the hospital setting. Specifically, there is limited information about pediatric hospital admissions, mortality patterns, and cause mortality in Ethiopian hospitals [6, 7]. Previous studies on pediatric hospital mortality in sub-Saharan Africa have demonstrated that deficiencies in pediatric triage, assessment, and emergency treatment at least partially accounts for high inpatient mortality, a large burden of which occurs early during hospitalization [8]. In Ethiopia, one study reported that 33 % of deaths occurred within the first 24 h of admission to the general pediatric ward [6], another reported 34 % within the first 48 h [7], while another reported 100 % of deaths within the first 24 h of admission to the pediatric ICU [9]. This suggests that a large proportion of pediatric hospital mortality occurs early during hospitalization, however available data is limited only to children less than five years of age and does not include a complete analysis of demographics, mortality patterns, and cause mortality. A better understanding of this information and an expanded epidemiological profile of admitted children in all age categories may aid in efforts to improve pediatric hospital triage, hospital resource allocation, and overall care [9].

Our aim was to examine the temporal relationship of mortality to admission, to describe the demographics of children in all age categories admitted to the Zewditu Memorial Hospital (ZMH) in Addis Ababa, and to identify the most common causes of hospital mortality.

## Methods

### Study setting

Our study was conducted in the pediatric emergency room and pediatric hospital ward at ZMH in Addis Ababa, Ethiopia. Addis Ababa is the capital city of Ethiopia. It is a rapidly growing, densely populated, urban city with a population of over three million people [10]. ZMH is a large teaching and referral hospital associated with Addis Ababa University that each year provides healthcare to approximately 7,000 pediatric patients aged 29 days – 14 years. It serves as a regional and referral hospital for children under the age of five, and a referral hospital for children ages 5–14. Patients are referred from clinics, health centers, and other hospitals, with all referrals admitted through the ZMH emergency department. ZMH has 41 pediatric beds and approximately 1,440 children are admitted to the pediatric ward annually. Neonates less than 29 days of age are cared for in a separate unit of the hospital and patients aged 15 years and older are admitted through the adult emergency room facilities.

Our study was conducted through a partnership between medical students and physicians of Addis Ababa University School of Medicine and Public Health and the University of Utah School of Medicine in the United States. Institutional Review Boards of the Addis Ababa Health Bureau, Addis Ababa University, and the University of Utah approved the study protocol.

### Study design

We conducted a four-year retrospective review of pediatric admissions at ZMH. Admission entries were documented in registration books in both the pediatric emergency room and hospital ward. Admission entries from 2011–2014 of children age 29 days-14 years were reviewed. Neonates less than 29 days of age were excluded from our analysis due to confounders relating to preterm birth and intrapartum-related complications [11]. Age, gender, admission date, disease classification, discharge status, and discharge date were obtained and recorded by investigators.

### Statistical analysis

Mortality rates were calculated by dividing the number of hospital deaths by the number of hospital admissions. Early mortality was defined as deaths occurring within two days of admission and is reported both as absolute early mortality rate and as a proportion of all deaths. Patient gender was compared using Chi-square analysis. A descriptive analysis was used for age and cause mortality.

## Results

ZMH recorded 6,866 entries between 2011 and 2014. Patient outcomes were stratified by age (Table 1). The proportion of admissions younger than five years of age was 0.747 (95 % CI, 0.736-0.757). Overall mortality for all reviewed records was 0.042 (95 % CI, 0.037-0.047), and early mortality was 0.018 (95 % CI, 0.015-0.022). The proportion of early deaths compared to total deaths was 0.437 (95 % CI, 0.380-0.494).

**Table 1:** Outcomes for pediatric patient entries at ZMH from 2011–2014 (*n* = 6866)

Age	Admission	Discharged Home N (%)	Deceased	Early Mortality^a^	Referred^b^	Left AMA	Unknown^c^
1–12 months	2657	1995 (75.1)	134 (5.0)	60 (2.2)	190 (7.2)	36 (1.4)	297 (11.2)
1 year	1132	881 (77.8)	46 (4.1)	18 (1.6)	78 (6.9)	11 (1.0)	116 (10.2)
2–5 years	1340	1054 (78.7)	44 (3.3)	21 (1.6)	75 (5.6)	19 (1.4)	148 (11.0)
6–14 years	1737	1397 (80.4)	59 (3.4)	27 (1.6)	97 (5.6)	18 (1.0)	166 (9.6)
Total	6866	5327 (77.6)	288 (4.2)	126 (1.8)	440 (6.4)	84 (1.2)	727 (10.6)

N = number of entries and (%) is the proportion of entries in each outcome by age. ^a^Early mortality defined as death occurring within 2 days of admission. ^b^Referred to another hospital. ^c^Unknown outcomes due to incomplete data for *n* = 727 patients

Sex identification was available for 6,821 patients (Table 2). The proportion of male admissions was significantly higher than female admissions in all age groups (male 0.575, *p* < 0.0001, 95 % CI 0.562-0.586).

**Table 2:** Sex identification from pediatric patient entries at ZMH from 2011–2014 (*n* = 6821)

	1–12 months N (%)	1 year	2–5 years	6–14 years	Deceased
Male	1509 (57.2)	658 (58.7)	743 (55.9)	1012 (58.5)	156 (54.2)
Female	1131 (42.8)	463 (41.3)	586 (44.1)	719 (41.5)	132 (45.8)

N = number of entries and (%) is the proportion of entries in each outcome by gender

For all reviewed records, the recorded diagnoses of deceased patients were pneumonia (0.253, 95 % CI, 0.203-0.303), severe acute malnutrition (0.222, 95 % CI 0.174-0.27), HIV/AIDS-related complications (0.056, 95 % CI 0.029-0.083), spina bifida (0.049, 95 % CI 0.024-0.074), and hydrocephalus (0.045, 95 % CI 0.021-0.069). These diagnoses were confirmed clinically by physicians; autopsies were not used.

## Discussion

Our study revealed a lower hospital mortality rate than has been previously reported in Ethiopia [6, 7, 12]. Despite this lower rate, 44 % of pediatric hospital deaths at ZMH occur early during hospitalization, which is higher than previously reported at other Ethiopian hospitals [6, 7]. High early mortality is likely multifactorial, related to patterns of severe disease, delays in care seeking by family members, inadequate pre-hospital care, and under-prioritized emergency care in hospital [13–15]. Improving pre-hospital emergency medical services (EMS) is emerging as a priority throughout low- and middle-income countries (LMIC) and has shown success at decreasing both mortality and long-term human and economic costs of illness and injury in other settings [16–18]. However, a coordinated EMS system in Ethiopia is not widely available [19, 20]. While a coordinated EMS system has a theoretical chance of decreasing early mortality, we did not collect data on time of injury or beginning of illness to time of hospital presentation, nor did we track method of arrival to hospital.

Our overall hospital mortality was not as high as reports from other African settings [1, 2, 21]. This is consistent with the trend of decreasing pediatric mortality in Ethiopia [1, 2]. Although a significant proportion of our hospital mortality occurred early during hospitalization, early mortality at ZMH is also lower than reports from other areas in the African region [8, 22, 23]. ZMH has a dedicated pediatric emergency care area that focuses on triage and rapid stabilization of patients upon arrival to the hospital. These elements of pediatric emergency care prioritization have been previously shown to decrease pediatric hospital mortality in Malawi and likely contribute to the lower than expected mortality and early mortality rate at ZMH [8]. Additionally, Addis Ababa does not have significant malaria transmission with the associated morbidity and mortality which accounts for a significant burden of acute illness and death in other hospitals in the African region which have reported on early hospital mortality patterns [24]. Yet even with the lower overall and early mortality rates, still close to half of all pediatric deaths at ZMH occurred within the first two days of admission, adding further evidence that systematic efforts and resources for pediatric emergency care should be prioritized in LMIC hospitals [8].

Pneumonia and severe acute malnutrition were the two main recorded causes of mortality in all age groups. This is consistent with other pediatric mortality studies in analogous settings [6, 7]. Ethiopia is one of several countries which suffers the largest burden of childhood mortality from pneumonia [25]. In fact, Ethiopia was included as one of six countries in Sub-Saharan Africa with high pneumonia mortality in an analysis to assess care-seeking behaviors related to respiratory illness, finding that only 30 % of Ethiopian children with suspected pneumonia were taken to a health care provider; the lowest of all six analyzed countries [26]. Other Ethiopian studies indicate that lack of knowledge, delay in recognition of illness severity, and household income are important factors that influence care-seeking behaviors [27, 28]. The HEP focuses on disease prevention and health education while providing antibiotic treatment for childhood pneumonia. However, challenges such as accuracy of diagnosis and appropriate referral to a health facility by the HEW remain barriers to further improvement [4, 29]. For respiratory illness as well as all acute pediatric illness, delayed care-seeking behavior, late recognition of illness severity by caregivers, and mismanagement of children with severe disease likely contributes to the high rates of early mortality and should be areas of focus and improvement for HEWs.

Finally, we found a significant male-to-female disparity in pediatric admissions to ZMH (0.57 male) despite equal sex population estimates for Ethiopian children ages 0–14 (0.50 male) [10]. Challenges with record keeping in LMIC hospitals have been well recognized [1, 30], however we have accurate sex identification for 99.4 % of our records, making this an unlikely contributor to the observed disparity. Sex disparity in hospital admissions has also been reported in other Ethiopian hospitals, implying that this may be a nation-wide phenomenon [6, 9]. It may reflect underlying societal factors affecting care-seeking behaviors by family members, and while this is beyond the scope of our analysis, it deserves further investigation. Early health interventions could potentially impact illness in children. An Ethiopian study did find that female infants were 20 % more likely to be breastfed than males, yet on its own this is unlikely to contribute to such a difference in admission [31]. Additionally, several studies report no relationship between sex and immunization rates in Ethiopia [32–34]. There are some limited data which suggest that respiratory illness may be more severe and diarrhea may be more prevalent in males in Ethiopia [35], but these studies primarily included neonates, a cohort not examined in this study. While we are unable to identify why there is such a marked sex disparity in pediatric hospital admissions to ZMH, it is clear that it exists and further investigation regarding social factors that influence care-seeking behaviors and early interventions are warranted.

We acknowledge that our study is limited by the use of handwritten medical records that are occasionally incomplete. Additionally, we recognize that medical recording varies amongst providers and introduces bias and that the cause of death made by the clinician may not accurately reflect the entire cause of death given that autopsy is not routinely performed. Despite these well-recognized limitations in resource-limited settings, we have included all data for which there are adequate records and believe this study may provide valuable information for clinicians and decision makers in similar clinical environments.

## Conclusion

Our study demonstrates a lower than expected hospital mortality at ZMH, however a large proportion of mortality still occurs early during hospitalization. Efforts to improve early care seeking by guardians for childhood illnesses, pre-hospital care, and emergency services in hospital will continue to improve outcomes among hospitalized Ethiopian children. Further work is also required to understand and address sex disparity as it relates to hospital admissions in Ethiopia.

## Abbreviations

CI: Confidence interval
EMS: Emergency medical service
HEP: Health extension program
HEW: Health extension worker
LMIC: Low- and middle-income countries
MGD: Millennium development goal
ZMH: Zewditu Memorial Hospital
